# Oncological Outcomes in Patients with Appendicular Myxofibrosarcomas: A Retrospective Study

**DOI:** 10.1155/2021/1844816

**Published:** 2021-11-28

**Authors:** Yonghoon Lee, Michael P. Guertin, Spencer Summers, Sheila A. Conway, Mothasem Al Maaieh, Raphael Yechieli, Jonathan Trent, Andrew E. Rosenberg, Juan Pretell-Mazzini

**Affiliations:** ^1^Miller School of Medicine, University of Miami, Miami, FL 33136, USA; ^2^Orthopedic Surgery Resident, Department of Orthopedic Surgery, Jackson Memorial Hospital, Miami, FL, USA; ^3^Musculoskeletal Oncology Division, Department of Orthopedics, Miller School of Medicine, University of Miami, Miami, FL 33136, USA; ^4^Director of Spine Tumors, University of Miami, Miami, FL 33136, USA; ^5^University of Miami, Jackson Memorial Hospital, Miami, FL, USA; ^6^Director of Anatomic Pathology, Director of Bone & Soft Tissue Pathology, Department of Pathology, Miller School of Medicine, University of Miami, Miami, FL 33136, USA; ^7^Director of Clinical Research Musculoskeletal Oncology Division, Musculoskeletal Oncology Division, Department of Orthopedics, Miller School of Medicine, University of Miami, Miami, FL, USA

## Abstract

**Background:**

Myxofibrosarcoma (MFS) is notorious for its infiltrative growth pattern, making wide excisions difficult to achieve. Our objective was to assess the impact of surgical margins and other factors that affected rates of local recurrence (LR), distant metastasis (DM), and overall survival (OS) of individuals undergoing resection for MFS.

**Methods:**

We retrospectively reviewed the medical records of 209 patients with appendicular soft tissue sarcomas between January 2012 and June 2018. Of these, 29 patients (14%) were diagnosed with myxofibrosarcoma. These patients underwent a total of 33 resections. The pathological analyses were conducted by an experienced musculoskeletal (MSK) pathologist. Demographics data, operative details, adjuvant therapy, and oncological outcomes were assessed.

**Results:**

Of the 29 patients (33 resections), the overall LR rate was 24% (7/29) and the 2-year LR rate was 17% (5/29). Factors associated with negative oncological outcomes were as follows: tumor size ≤10 cm (2-year local recurrence-free rates (LRFRs), 65%; 95% CI, 44–86%; *p*=0.02) and positive surgical margins grouped with surgical margins ≤0.1 cm (hazard ratio (HR), 11.74; 95% CI, 1.41–97.74; *p*=0.02). Chemotherapy and radiotherapy together increased the 2-year LRFR (LRFR, 100%; 95% CI, 100%, *p*=0.001). Two-year DM and OS rates were 15% and 79%, respectively. Female gender was a predictor of distant metastasis. Local recurrence had a negative impact on overall survival. Intraoperative analysis of resection margin accuracy was 75% (12/16) when non-MSK pathologists were involved but 100% accurate (12/12) when analyzed by an MSK pathologist.

**Conclusion:**

Myxofibrosarcomas showed high LR rates after treatment. Close margins (≤0.1 cm) should be considered as a risk factor for LR, and LR is associated with negative overall survival. Neoadjuvant therapy in terms of combined chemotherapy and radiation therapy associates with decreased LR rates. If intraoperative assessment of margins is to be done, it should be performed by an experienced MSK pathologist.

## 1. Introduction

Myxofibrosarcoma (MFS) is a soft tissue sarcoma encompassed with malignant fibroblastic lesions with irregular myxoid stroma. This rare malignant tumor is most commonly seen in the elderly, 60–80 years old, with a propensity of forming in extremities [1]. Myxofibrosarcomas tend to have a higher local recurrence (LR) rate (32%–60%) when compared to other soft tissue sarcomas, which might be explained by its infiltrative characteristics [2–5]. MRI findings depict myxofibrosarcomas with a distinctive pattern of diffuse spreading along well-defined and tapering fascial boundaries [6, 7]. This aggressive and subtle growth makes diagnosis and proper initial surgery of upmost importance for better prognosis [8].

Surgical resection is the treatment of choice for MFS. Its infiltrative growth pattern highlights the importance of wide margins on surgical excision as the literature has shown that positive surgical margins and narrow margins (e.g., ≤0.1 cm) are associated with increased risks of LR, distant metastasis (DM), or lower overall survival (OS) rate [9–12]. When resection was required for local recurrent tumors that were previously resected from other outside facilities, the prognosis is typically poorer [6, 8].

Existing research has examined a variety of elements that are associated with LR, DM, and OS rates of MFS patients. Studies have exhibited findings that older age (>65 years) is associated with increased LR rates and decreased OS [9, 10]. Specific tumor characteristics can have effects on these variables. Larger tumor size and higher grading have been shown to associate with poorer survival [11, 13]. Aside from the health difficulties that accompany cancer, a previous examination showed both higher grading and the need for recurrent surgical resection have increased needs for amputation [14]. Consequently, elderly individuals who have undergone amputation tend to live at a less functional level [15]. As such, the effects of a poor surgical margin can become a determinant in lifestyle and life expectancy.

Adjunctive and neoadjunctive therapies often accompany surgical treatment for MFS. To our knowledge, there are no consistent findings that make a certain combination of therapies more likely to reduce LR rates. Some studies have concluded that there is no significant relationship between decreased LR and combinations of radiotherapy and chemotherapy [6, 11, 16], nor has there been ample analysis on the effects of these adjunctive therapies for MFS patients [8].

Due to the infiltrative growth pattern of MFS tumors, intraoperative frozen sections are often assessed by a pathologist to ensure margins are negative. While the frozen section has an overall diagnostic accuracy ranging from 89 to 98% [17, 18], the diagnostic accuracy of frozen section analysis in the setting of MFS has never been directly evaluated. Given that positive margins can occur in up to 20% of MFS resections [9, 14], knowing the diagnostic accuracy of the frozen section analysis is critical.

Therefore, we asked the following: (1) What is the local recurrence rate after surgical treatment of myxofibrosarcomas in a sarcoma center? (2) Which factors are associated with local recurrence rate? (3) What is the distant metastatic and overall survival rate in patients affected by this tumor, and what are the contributing factors? (4) What is the accuracy of intraoperative assessment of surgical resection margins?

## 2. Materials and Methods

A retrospective review of medical records of 209 patients with appendicular soft tissue sarcomas between the years 2012 and 2018 was performed after approval by the Institutional Review Board of our institution. Patients with <2 years of follow-up data were excluded from the study. Of these, 29 patients (14%) were diagnosed with myxofibrosarcoma with a total of 33 resections. The pathological analyses were conducted by an experienced musculoskeletal pathologist.

This study analyzed the demographic variables: age, gender, race, stage, tumor location (upper vs lower extremity), and laterality (left vs right); tumor characteristics: grading, depth (subcutaneous vs intramuscular), size, necrosis, and margin status (negative (≤0.1 cm, 0.1–0.49 cm, ≥0.5 cm) vs positive); adjuvant therapy: radiation therapy and chemotherapy; oncological outcomes: LR rate and OS; and accuracy of intraoperative analysis of resection margins. Demographics were obtained from medical records and tumor characteristics were obtained from the final pathology report.

**Figure 1:**
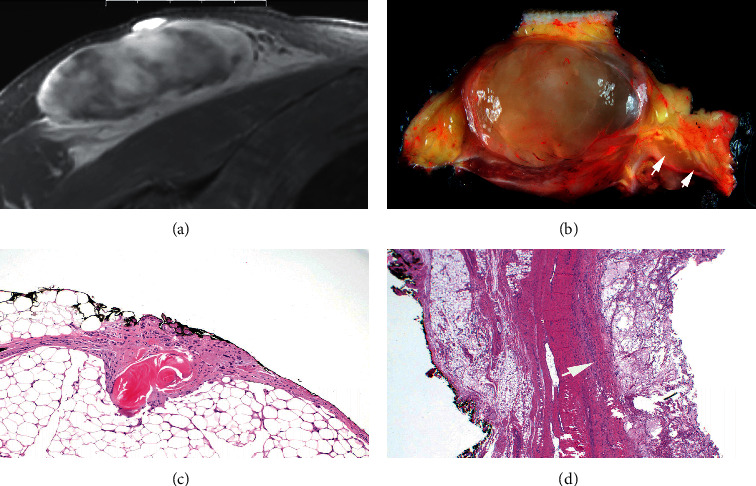
(a) Ax-T2-MR demonstrating tumor with infiltrating margins in subcutaneous fat. (b) Tumor in subcutis with infiltrative tentacles (arrows). (c) Tumor with malignant spindle cells infiltrating fat, present at the ink surface. (d) Negative margin with tumor confined by pseudocapsule (arrow).

Grading was determined via the French Federation of Cancer Center Sarcoma Group, with tumor differentiation (well differentiated to undifferentiated), mitotic count (0–9, 10–19, to 20 or more), and necrosis (none, <50%, to >50%) for a grade from 1 to 3. The largest dimension of the tumor was picked as the representative tumor size. Margin status was classified as negative if the inked margins had no cells that were considered malignant (Figures 1(a)–1(d)). For chemotherapy, adriamycin-based systemic therapy was done for 2 cycles for adjuvant therapy while 2–4 cycles were done for neoadjuvant therapy for a total of 6 cycles. Fifty grays of radiation were given as neoadjuvant and 66 Gy as adjuvant.

Intraoperative analyses of margins were determined to be accurate if the intraoperative and final pathology report came to the same conclusion; i.e., both reports determined the resected margins to be negative or positive. Analyses were considered inaccurate if the intraoperative report stated negative margins, but the final report stated positive margins, or vice versa.

All surgeries and neo/adjuvant therapies were performed at one of two hospitals.

### 2.1. Statistical Analysis

Local recurrence-free rates, metastatic-free rates, overall survival rates, and factors associated with these rates were examined. Kaplan–Meier analysis was used to determine the rates with time zero defined as the date of operation and censored if no event occurred two years after resection. Local recurrence (LR), metastasis, or death was counted as an event if it happened within two years of resection. The log-rank test was used to determine significant differences in survival times between factors. Probability values < 0.05 were considered significant.

Hazard ratios (HRs) were calculated via univariate Cox regression to determine predictors of LR and OS. Spearman's correlation was used to compare the relationship between variables. Multivariate analysis was not performed due to the small cohort size and lack of statistical power. Statistical analysis was performed using SPSS 26.0 software (IBM Corp, New York, NY, USA).

## 3. Results

### 3.1. Patients and Tumor Characteristics

In total, 29 patients with myxofibrosarcoma constituted our study population. Patient and disease characteristics are listed in Table 1. The mean age of the patients was 68 years (range 46–90 years); there were 17 females and 12 males. All patients were treated with surgical resection.

Of the 33 tumors resected, 15% of tumors were intermediate grade and 85% were high grade. Tumor size was either considered ≤10 cm (67% of patients) or >10 cm (33%). Seventy-six percent (25/33) were wide resections, and 24% (8/33) were amputations. Forty-six percent (6/13) of tumors larger than 10 cm underwent amputations or disarticulations, while only 11% (2/18) of tumors less than 10 cm were amputated. High-risk tumors, those larger than 10 cm, were significantly more likely to be amputated rather than limb-sparing surgery (Spearman's rho = 0.39, *p*=0.025). Twenty-one percent of resections had positive margins (7/33), and 79% (26/33) had negative margins. Of the resections with positive margins, 71% were tumors located in the subcutaneous tissue (71% vs 29% intramuscular, *p*=0.23) and all of them showed an infiltrative pattern of growth.

When the negative margins were further categorized, 24% had malignant cells ≤0.1 cm from the inked margins and 55% had cells >0.1 cm from the tumor border. Tumor depth was divided into subcutaneous (52%) and intramuscular (48%). The tumor pattern of growth was divided into whether it was well-circumscribed (55%) or infiltrative (45%).

### 3.2. Treatment

Seventeen (52%) cases were treated with a combination of radiotherapy and chemotherapy, 8 (24%) with radiotherapy alone, 4 (12%) with chemotherapy alone, and 4 (12%) with neither radiotherapy nor chemotherapy. Patient follow-up time ranged from 0.36 to 6.63 years (median, 3.38 years).

### 3.3. Local Recurrence

**Figure 2:**
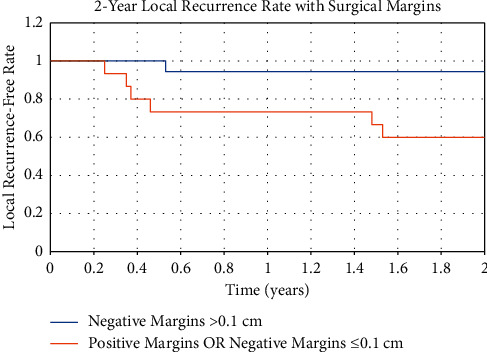
Figure showing the 2-year local recurrence-free rate comparing patients with negative margins >0.1 cm and patients with either negative margins <0.1 cm or positive margins.

The overall LR rate of patients was 24% (7/29), and the 2-year LR rate was 17% (5/29). Of the five that had a LR within two years, one experienced multiple recurrences. Median time to first LR was 1.8 years (range, 0.35–6.31 years). Using the log-rank test, factors associated with lower local recurrence-free rates (LRFRs) were tumor size ≤10 cm (2-year LRFR, 65%; 95% confidence interval (CI), 44–86%; *p*=0.02) and surgical resection margins ≤0.1 cm (2-year LRFR, 63%; 95% CI, 29–97%; *p*=0.033) (Table 2). Resections were grouped with positive and negative margins ≤0.1 cm together and showed a more significant decrease in LRFRs (2-year LRFR, 60%; 95% CI, 34–86%; *p*=0.015) (Figure 2).

Chemotherapy and radiotherapy together (2-year LRFR, 100%; 95% CI, 100%, *p*=0.001) had an association with higher LRFRs (Table 2). Categorical variables were analyzed for hazard ratios with a Cox regression unless no events occurred in the sample. Because patients with tumor size >10 cm and those who received both radiation and chemotherapy experience no local recurrences, only the Kaplan–Meier log-rank test was used to assess statistical significance of these LRFRs.

Using univariate Cox regression analysis, positive surgical margins grouped with negative margins ≤0.1 cm were a factor predictive of LR (hazard ratio (HR), 11.74; 95% CI, 1.41–97.74; *p*=0.02). Even though there was no statistical significance, subcutaneous location (29% (5/17) vs 13% (2/16), *p*=0.2) showed a tendency for higher LR rates.

**Figure 3:**
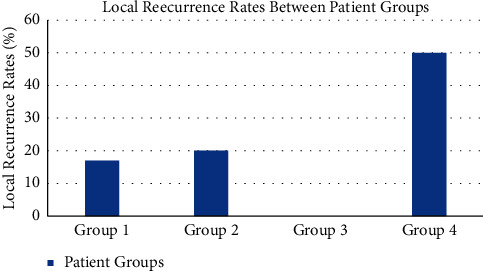
2-year local recurrence rates (LRRs) compared between (1) all patients, (2) a second recurrence in patients who had a LR, (3) patients with a re-resection after positive final pathology reports, and (4) patients without a re-resection after positive final pathology reports.

Re-resections were classified as either a subsequent resection after a positive intraoperative report or a resection done postoperatively within a month of a positive final pathology report. Re-resections after a positive intraoperative report showed a tendency for higher LR rate (2/4) compared to re-resections done postoperatively after a positive final pathology report (0/4) (*p*=0.19) (Figure 3). Conversely, the latter LR rate (0/4) was notably different when compared to resections after a local recurrence (3/7) (*p*=0.07).

### 3.4. Distant Metastasis and Survival

Both overall and the two-year DM rates were 21% (6/29). Median time to DM was 0.80 years (range, 0.19–1.93 years). The metastatic sites included the breast [1], lungs [2], thigh [1], brain [1], and thyroid [1]. Using the log-rank test, the only patient demographic or tumor variable associated with lower metastasis-free rate (MFR) was female gender (2-year MFR, 58%; 95% CI, 30%–86%; *p*=0.014). Using Cox regression analysis, it was also a predictive factor of DM (HR, 9.2; 95% CI, 1.07–79.1; *p*=0.04) (Table 3).

The two-year OS rate was 79% (23/29). The overall OS rate was 72% (21/29). Of the categorical variables measured with the log-rank test, LR was the only significant factor with a negative impact on OS rate (2-year OS rates, 50%; 95% CI, 10%–90%; *p*=0.02). Using Cox regression, it was also a predictive factor of OS (HR, 6.36; 95% CI, 1.06–38.20; *p*=0.04) (Table 4).

### 3.5. Intraoperative Accuracy

Intraoperative analysis of resection margin accuracy was 75% (12/16) when non-MSK pathologists were involved; however, when the specimen was analyzed by an MSK pathologist, accuracy was 100% (12/12). In every case of a false negative, the margins that the pathologist examined were negative, but malignancy was detected in other parts of the specimen that were not examined at the time of the operation. All intraoperative resections were reviewed postoperatively by an MSK pathologist for the final report.

## 4. Discussion

### 4.1. Local Recurrence

There was a 24% (7/29) LR rate after resecting a tumor, and 17% (5/29) of patients experienced LR within 2 years. Previous studies have reported similar LR rates of MFS [9, 11, 14]. Our LR rate was lower than that presented in the study by Mentzel et al. where they identified a 54% LR rate [3], while Ghazala et al. reported a lower LR rate than ours, 14% [19], showing heterogeneity in the data. An important distinction to note between studies is the interval of recurrence as many studies used variable intervals from 2 to 10 years to examine LR rates.

### 4.2. Factors Associated with Local Recurrence

This study showed that positive and negative margins ≤0.1 cm were a significant predictor of LR. In previous studies, positive and close margins have had a significant association with LR. For MFS resections, Grimer et al. found high LR rates (63%) in high-grade tumors after re-resections of soft tissue sarcomas with positive margins [20]. Historically, close margins have consisted of a small range of margin sizes, usually ≤1.0 cm [9, 11, 14]. To narrow this marginal range, we grouped negative margins as ≤0.1 cm, 0.1cm–0.49 cm, and ≥0.5 cm. Only margins with cells >0.1 cm from the tumor border demonstrated significant lowering of the LR rate. Thus, with respect to LR, close surgical margins ≤0.1 cm (38%, 3/8) are more equivalent to positive surgical margins (43%, 3/7) than to negative surgical margins >0.1 cm (6%, 1/18).

While this study showed that tumors >10 cm had smaller LRFRs, this does not correlate appropriately with previous findings. In fact, studies have shown a correlation that larger tumor sizes were a positive predictor of LR [21, 22]. Increasing tumor size has also been positively associated as a predictor for DM and decreased OS [9, 11, 16]. It is unclear why this study's findings show a different outcome compared to others. A possible explanation is that in this cohort, patients with tumors >10 cm were significantly more likely to undergo amputation or disarticulations, which appears to have provided better local control [23].

Interestingly, those who had a re-resection during the operation had a higher chance of recurrence within 2 years (2/4) compared to those who had a re-resection postoperatively (0/4). While these sample sizes are not large, these differences might stem from how the surgeon approached the different positive pathology reports. With a positive intraoperative report that is not comprehensive and with invasive MFS growth tendency, additional resections from the surgical bed might not be completed in accurate locations. This could be related to the experience of the pathologists assessing the specimen as we discuss later. However, after a positive final pathology report, there tends to be more detailed information on the specimen and margin specifications. This can lead to wider margins in a future surgery to ensure that the patient will not need a third operation if the re-resection is unsuccessful. Of note, all the final pathology assessments were done by the same experienced MSK pathologist.

O'Donnell et al. reported that unexpected positive margins around the soft tissue occur most frequently when a proposed surgical boundary such as fascia is not a true barrier to tumor spread or when there is an incorrect assessment of the peripheral cancer cells surrounding the tumor [7]. These can indicate that the tumor is more infiltrative and aggressive than expected, causing a higher risk of local recurrence. In our study, 71% of resections with positive margins were in the subcutaneous tissue and all had an infiltrative pattern of growth based on histologic analysis, indicating a higher risk of local recurrence.

A combination of radiotherapy and chemotherapy was a negative predictor of LR compared to only using radiotherapy, chemotherapy, or no therapy. Other studies have found varying conclusions with respect to the use of radiotherapy and/or chemotherapy. Some studies showed inconclusive evidence of the effectiveness of radiation and chemotherapy as an adjuvant to resection [6, 8, 11]. Another study discussed the benefits of radiotherapy for local control, regardless of whether chemotherapy was also used or not [16]. Moreover, no systematic differences in LR rates were found with respect to the timing of radiotherapy, whether it was neoadjuvant or adjuvant therapy [9, 16, 24]. These inconclusive findings may be explained due to smaller samples of patients receiving none or one type of adjuvant therapy, making it difficult to statistically compare treatments of the combined modalities. In these present findings, it is noteworthy that none of the seventeen patients treated with both radiotherapy and chemotherapy had a LR. Therefore, when considering treatments and the patients' clinical circumstances, it may be in their best interest to receive both forms of adjuvant therapies.

### 4.3. Distant Metastasis and Overall Survival: Rates and Contributing Factors

The 21% DM rate in this study is similar to the rates in previous investigations [9, 10, 14, 16]. The same is true with respect to the 2-year OS rate of 79% [9, 10, 14].

While MFS tends to predominate in males [1], female gender was shown to be a predictor of DM. To our knowledge, other studies have not reported gender as a predictor of metastasis, so this unique finding will be further investigated in future studies.

Other studies identified large tumor sizes (>5 cm) as a risk factor for DM, which was not found in our study [9, 11]. Again, this may be due to the aggressive surgical treatments, namely, amputations, that could provide better control of the tumor. As discussed in the literature, this study concurs that LR is a negative predictor of OS. A multitude of variables may be responsible for this poorer prognosis. Recurrence can indicate a more aggressive, infiltrating tumor, which may have a higher probability of metastasizing, thereby decreasing chances of survival [25, 26]. While this study did not reach a similar conclusion, other studies showed that increasing age and tumor size were negatively associated with OS [9, 14].

### 4.4. Intraoperative Surgical Margin Assessment Accuracy

The literature describes that failure of the primary surgical resection to achieve adequate negative margins can be a predictor of poorer prognosis [6, 8, 27]. The infiltrative nature of MFS as seen in histological observations and MRI findings [1, 6, 7, 28] demonstrates the difficulties of obtaining adequate negative margins. Given the importance of accurate MFS diagnosis and surgical resection margins, an intraoperative assessment might be a necessary step. In this present study, intraoperative assessments done by non-MSK pathologists were less accurate than those performed by an MSK pathologist. Intraoperative reports might serve an integral purpose, but they are reliant on the pathologist's experience to be utilized effectively. Therefore, our findings suggest that MSK pathologists should be examining these surgical specimens to prevent inaccuracies and prevent re-resections due to false negatives.

### 4.5. Limitations

This study is subject to several limitations. First, the small number of patients makes statistical analysis of prognostic factors difficult. Factors that did not reach statistical significance in our analyses may become significant with a larger sample size. Second, there was a heterogeneity in tumor management as each patient's different circumstances brought about subjective decisions on the type of resection or adjuvant therapy. The number of cycles of chemotherapy is an example of a factor that varied from patient to patient. Third, due to loss of follow-up or death, the LR rate may be underestimated. Lastly, longer follow-up could change these findings and increase the rates of LR, DM, and decrease OS.

## 5. Conclusion

Myxofibrosarcomas show high LR rates after surgical treatment. Positive and negative margins ≤0.1 cm should be considered as a risk factor for LR, and intraoperative re-resections of the surgical bed may not decrease this risk. Neoadjuvant therapy in terms of combined chemotherapy and radiation therapy seems to decrease LR rates. Female gender is a significant predictor of DM. The OS at 2 years is 79% and is negatively affected by LR. Moreover, if intraoperative assessment of margins is to be done, it should be performed by an experienced MSK pathologist. Although these findings need to be confirmed in larger studies, the effective margin distance, the appropriate therapeutic modalities, and the necessity of utilizing MSK pathologists should be considered.

## Figures and Tables

**Table 1:** Demographics and tumor descriptions for each patient.

Patient ID	Gender	Race	Age	Location, left/right	Location, upper/lower	Tumor depth	Tumor character	Tumor grade	Tumor stage	Total follow-up time (years)
1	F	W	57	Left	Lower	SQ	Inf	3/3	Stage IIIA	2.32
2	M	W	74	Right	Lower	IM	WC	3/3	Stage II	5.73
3	M	W	66	Left	Lower	SQ	Inf	3/3	Stage IIIB	3.38
4	M	H	77	Right	Upper	IM	WC	3/3	Stage IIIA	5.82
5	M	W	56	Left	Lower	SQ	WC	3/3	Stage IIIB	5.56
6	F	W	64	Left	Lower	SQ	WC	3/3	Stage IIIB	2.47
7	F	W	67	Right	Lower	IM	WC	3/3	Stage IIIA	4.08
8	M	AA	46	Left	Lower	IM	WC	3/3	Stage II	2.78
9	F	W	82	Left	Upper	IM	Inf	2/3	Stage IIIB	5.12
10†	M	W	90	Left	Lower	SQ	WC	3/3	Stage IV	2.51
11	F	AA	65	Right	Lower	SQ	WC	3/3	Stage IIIB	1.3
12	M	W	54	Right	Lower	SQ	Inf	3/3	Stage IIIB	2.34
13	F	As	54	Left	Lower	SQ	WC	3/3	Stage II	6.25
14	M	W	66	Left	Lower	SQ	Inf	3/3	Stage IIIB	1.85
15	F	W	83	Left	Lower	IM	WC	3/3	Stage IIIB	6.63
16	M	AA	62	Right	Upper	IM	WC	2/3	Stage II	3.38
17	F	W	53	Left	Lower	IM	WC	2/3	Stage IIIB	3.75
18	M	W	59	Left	Lower	IM	Inf	3/3	Stage II	6.35
19	F	W	88	Left	Lower	IM	WC	2/3	Stage IIIB	3.82
20	M	W	47	Left	Lower	SQ	Inf	3/3	Stage IIIA	5.79
21	M	W	47	Left	Lower	SQ	Inf	3/3	Stage II	5.84
22	M	AA	67	Left	Lower	IM	WC	3/3	Stage IIIB	5.5
23	M	W	78	Right	Lower	IM	Inf	3/3	Stage IIIB	0.36
24^*∗*^	F	W	85	Left	Lower	IM	Inf	3/3	Stage II	3.02
25	F	W	64	Left	Lower	IM	WC	3/3	Stage II	2.91
26	F	W	67	Right	Lower	IM	WC	3/3	Stage II	3.66
27	M	AA	46	Left	Lower	IM	WC	3/3	Stage II	2.78
28	M	W	55	Right	Upper	SQ	WC	3/3	Stage II	2.42
29	M	W	63	Right	Lower	SQ	Inf	2/3	Stage IIIB	2.12

*Note. M* = male; *F* = female; *W* = white; AA = African American; *H* = Hispanic; As = Asian; SQ = subcutaneous; IM = intramuscular; Inf = infiltrative; WC = well-circumscribed; ^*∗*^ = one additional resection; ^†^ = three additional resections.

**Table 2:** Relationship between oncological outcomes and 2-year local recurrence-free rates.

Oncological outcomes		2-year LR over total	2-year LRFR (%)	HR	95% CI	*p* value
Tumor type	Myxofibrosarcoma	7/33	79			
Gender	Male	4/17	76	2.33	0.24–22.44	0.46
	Female	1/12	92			

Age	≤60 years old	1/11	91	0.50	0.05–4.80	0.55
	>60 years old	4/18	78			

Tumor size	≤10 cm	7/20	65	N/A^*∗∗*^	44–86%^*∗∗*^	0.02^*∗∗*^
	>10 cm	0/13	100			

Margin size	Negative ≤0.1 cm	3/8	63	7.97	0.83–76.79	0.07
	Negative >0.1 cm	1/18	94			

Surgical margins	Positive	3/7	57	—	—	—
	Close margins^*∗*^	3/8	63	0.95	0.19–4.73	0.95
	Negative >0.1 cm	1/18	94	0.11	0.01–1.07	0.06

Grouped surgical margins	Positive or close^*∗*^	6/13	54	11.74	1.41–97.74	0.02
	Negative >0.1 cm	1/20	95			

Tumor depth	Subcutaneous	5/17	71	2.56	0.50–13.21	0.26
	Intramuscular	2/16	88			

Tumor character	Well-circumscribed	2/18	89	0.30	0.06–1.54	0.15
	Infiltrative	5/15	67			

Re-resection intraoperatively†	Re-resection done	2/4	50	N/A^*∗∗*^	1–99%	0.19^*∗∗*^
	Re-resection not done	0/3	0			

Re-resection postoperativelyˆ	Re-resection done	0/4	100	N/A^*∗∗*^	100%^*∗∗*^	0.07^*∗∗*^
	Re-resection not done	2/3	67%			

Adjuvant therapy	Chemotherapy and radiotherapy	0/17	100%	N/A^*∗∗*^	100%^*∗∗*^	0.001^*∗∗*^
	Chemotherapy alone	3/4	25%			
	Radiotherapy alone	2/8	75%			
	No treatment	2/4	50%			

*Note.* LRFR = 2-year local recurrence-free rate; LR = local recurrence; HR = hazard ratio; CI = confidence interval. ^*∗*^Close margins = negative margins ≤0.1 cm; ^*∗∗*^the Kaplan–Meier log-ranked test was used to assess statistical significance; †re-resections were done intraoperatively due to positive intraoperative pathology report; ˆre-resections were done postopearatively due to positive final pathology report.

**Table 3:** Relationship between oncological outcomes and 2-year metastatic-free rates.

Oncological Outcome		2-year MR over total	2-year MFR	HR	95% CI	*p* value
Gender	Male	1/17	94%	9.2	1.07–79.16	0.043
	Female	5/12	58%			

*Note.* MR = metastatic rate; MFR = metastatic-free rate; HR = hazard ratio; CI = confidence interval.

**Table 4:** Relationship between oncological outcomes and 2-year overall survival rates.

Oncological outcome		Kaplan–Meier log-rank test			Cox regression		
		2-year SR over total	2-year OSR (%)	*p* value	HR	95% CI	*p* value
Recurrence	Yes	3/6	50	0.02	6.36	1.06–38.21	0.043
	No	2/23	91				

*Note.* SR = survival rate; OSR = overall survival rate; HR = hazard ratio; CI = confidence interval.

## Data Availability

The data used to support the findings of this study are available from the corresponding author upon request.
